# Tropical Secondary Forest Management Influences Frugivorous Bat Composition, Abundance and Fruit Consumption in Chiapas, Mexico

**DOI:** 10.1371/journal.pone.0077584

**Published:** 2013-10-11

**Authors:** Ivar Vleut, Samuel Israel Levy-Tacher, Willem Frederik de Boer, Jorge Galindo-González, Luis-Bernardo Vazquez

**Affiliations:** 1 Biodiversity Conservation, El Colegio De La Frontera Sur (ECOSUR), San Cristóbal de Las Casas, Chiapas, México; 2 Resource Ecology Group, Wageningen University, Wageningen, The Netherlands; 3 Instituto de Biotecnología y Ecología Aplicada (INBIOTECA), Universidad Veracruzana, Xalapa, Veracruz, México; University of Western Ontario, Canada

## Abstract

Most studies on frugivorous bat assemblages in secondary forests have concentrated on differences among successional stages, and have disregarded the effect of forest management. Secondary forest management practices alter the vegetation structure and fruit availability, important factors associated with differences in frugivorous bat assemblage structure, and fruit consumption and can therefore modify forest succession. Our objective was to elucidate factors (forest structural variables and fruit availability) determining bat diversity, abundance, composition and species-specific abundance of bats in (i) secondary forests managed by Lacandon farmers dominated by *Ochroma pyramidale*, in (ii) secondary forests without management, and in (iii) mature rain forests in Chiapas, Southern Mexico. Frugivorous bat species diversity (Shannon *H*’) was similar between forest types. However, bat abundance was highest in rain forest and *O. pyramidale* forests. Bat species composition was different among forest types with more *Carollia sowelli* and *Sturnira lilium* captures in *O. pyramidale* forests. Overall, bat fruit consumption was dominated by early-successional shrubs, highest late-successional fruit consumption was found in rain forests and more bats consumed early-successional shrub fruits in *O. pyramidale* forests. *Ochroma pyramidale* forests presented a higher canopy openness, tree height, lower tree density and diversity of fruit than secondary forests. Tree density and canopy openness were negatively correlated with bat species diversity and bat abundance, but bat abundance increased with fruit abundance and tree height. Hence, secondary forest management alters forests’ structural characteristics and resource availability, and shapes the frugivorous bat community structure, and thereby the fruit consumption by bats.

## Introduction

Due to the rapid conversion of rain forest into agricultural fields and secondary forest, with a deforestation rate of 0.5%/year, secondary forests will occupy a high percentage of the total forested area in the world in the next decades [1,2]. However, little evidence is available on the impact of secondary forest management strategies on frugivorous bats. Bats play a key role in forest regeneration processes, they are important contributors of propagules, facilitate the reproductive success of plants, and play an important role in the economic value of forests [3,4], as 70-98% of flowering woody plants from rain forest are dispersed by bats and other vertebrates [5–7]. 

The Mexican Maya Lacandon, developed effective management strategies for the fallow periods [8–10], which includes the sowing of selected tree species during this period, such as *Swietenia macrophylla* or *Ceiba pentandra* for their timber qualities, or *Ochroma pyramidale*, a pioneer tree known for its rapid growth and capacity to restore soil fertility [11]. *Ochroma pyramidale* is a fast growing light demanding early-successional species [9,12–14], able to reach up to 6 m in a year, that can improve soil fertility through the acceleration of soil organic matter accumulation [15,16]. Changes in structure and composition due to specific traits of this dominant tree could trigger important cascading effects, as compositional and structural differences in tropical forests have been associated with differences in the bat species composition or species-specific abundance [17–21]. *Ochroma pyramidale* secondary forests presented higher canopy openness and tree height, lower tree density and higher shrub density in the understory in comparison with control secondary forests [11]. A higher canopy openness could negatively affect bat assemblage, due to increasing predation risk [22], but could increase the abundance of *Carollia* and *Sturnira* bats, preferring fruits from light demanding early-successional shrub species (e.g. Piperaceae and Solanaceae) [7,20,21,23,24]. 

To test this, we first compared bat species diversity, bat abundance, composition and the abundance of the most dominant bat species among three forest types: managed secondary forest (with *O. pyramidale*), control secondary forests (without management) and rain forests. Secondary, we evaluated environmental variables, divided into structural variables (canopy openness, tree density, height and diversity) and fruit availability variables (diversity and abundance) among forest types, and correlated these variables with the frugivorous bat diversity, bat abundance and species-specific responses. 

We expected to find that: (i) a different bat species composition among forest types with a higher abundance of bat species associated with open canopies, such as *Carollia* and *Sturnira*, in managed secondary forest with *O. pyramidale* compared to control secondary forests and rain forests (ii) the species composition of consumed fruits differs among forest types and more bats consume fruits of early-successional shrub species in the more open *O. pyramidale* forests in comparison with control secondary forests and rain forests (iii) bat diversity and abundance increases with decreasing tree density and increasing tree height.

## Materials and Methods

The bats were captured with a Scientific Collector’s permit (Colector Científico de Flora y Fauna) issued by the environmental authority in Mexico (SEMARNAT permit number 04787/10). The guidelines of investigating flora and fauna in the Mexican territory conform to the policies of the Division of Graduate Studies and the Ethics Committee of El Colegio de la Frontera Sur. They approved the research study on “Factors that determine the presence of bats in secondary forests in the Maya Lacandon community Lacanhá, Chiapas, Mexico” to the first author on July, 2009. The bats were captured using mist nets and released after identifying and measuring bat individuals. None of the captured individuals were sacrificed. 

### Study sites

We studied 12 sites in the Maya Lacandon community in Chiapas, Mexico (16° 46’ 08” N, 91° 08’ 12” W); eight sites were secondary forest patches of 0.5-1.0 ha extent and the other four were sites in the matrix of rain forest (1.0 ha). The community of Lacanhá is located on the margin of Montes Azules Biosphere Reserve, Chiapas, Mexico with an elevation of 355-370 m above sea level [25] and a dominating surface of rain forest (Maldonado Unpublished data). For a more detailed view of the study area and the locations of the study sites see Vleut et al. [11]. All study sites were located on private lands and owners Manuel Castellano and Eva Chankin gave consent to conduct the study on their lands. The secondary forest patches were cultivated using slash-and-burn, and abandoned 10-15 years ago. The eight secondary forest patches were divided over two treatments: four patches of secondary forest were dominated by the pioneer tree *O. pyramidale* (Malvaceae), hereafter called *O. pyramidale* forests, product of management by Lacandon farmers before the end of the cultivation period; and four patches of secondary forests without any preference for tree species (hereafter called secondary forests), so we have three treatments (*O. pyramidale* forests, secondary forest and rain forest) with four replicates each. The proportion of surrounding rain forest around secondary forest patches was similar among sites to reduce the effect of confounding variables on bat diversity [21].

### Bat sampling

Bats were captured each month using 3 (12.0 x 2.4 m, 36 mm mesh) mist nets per site, set at ground level, starting 0.5 h before sunset until 4h after, one night per site, from August 2010-July 2011. This study focused on phyllostomid bats only, and the techniques we used are widely accepted as effective sampling methods for these bats [18,26]. The total sampling effort was 74,650 m^2^·h [27]. Trails, roads and rivers were avoided while capturing bats, for their potential bias towards bat species [28]. Vegetation structure can differ between the edges in comparison with the interior of the forests and possibly affecting affect bat assemblage. The influence of the proximity towards the boundary is significantly reduced beyond 15-25 m into the forest and we therefore placed mist nets at least 25 m from the border of sites [29]. Bats were not captured during and 2 days prior and after a full moon. In the case of heavy rain we waited until the rain passed, shook excess of the mist nets and resumed counting the 4 and a half hour of bat sampling following Medellín et al. [17]. Captured bats were identified using field guides [30,31]. Before releasing at the capture site, the forearm of each individual was marked with a colored marker, to avoid recounting during the same night. 

### Fruit consumption

Although frugivorous bat are considered to specialize in the consumption of fruits, they supplement their diet with other nitrogen or protein rich sources such as insects, foliage or nectar [32–34]. However, during this manuscript we focus on the consumption of fruits by bats by either determining the fruit species from the collected fecal samples or by the fruits carried by the bats. Carried fruits can include fruits with larger sized seeds which are not swallowed by the bat, but are dispersed when the pulp is consumed. 

Fruit consumption data from fecal samples was gathered by collecting seeds from plastic sheets placed below the mist nets or from cloth bag in which the bats were individually held for a period of 30 min [35]. In the case of overlapping bat individuals of different species in the mist nets we excluded the fecal samples on the plastic sheets from the database, to avoid assigning the fecal samples to the wrong species. The collected fecal samples were stored, dried and kept in small paper bags, and seeds were then separated from fruit pulp, insects and other fecal remains. Carried fruits and seeds from fecal samples were identified to species level when possible. Fruit consumption was quantified by the number of different fruit species carried or defecated per bat per site. We categorized fruit consumption into life form and successional stage; life form and successional stage comprised two classes, respectively shrubs or trees, and early-successional stage (light demanding plant species) or late-successional species (shade tolerant plant species), based on tolerance to shade and seed size [36,37]. 

### Forest structure and fruit availability

Canopy trees were measured in six randomly selected quadrats of 10 × 10 m per site in which dbh (diameter at breast height) and height of all trees ≥5 cm dbh (diameter at breast height) were measured and identified. The mean tree density, height and diversity (Shannon *H*’) was estimated per site as well as the percentage of canopy openness at 15 random points using a hemispherical crown densiometer (Forestry Suppliers, Inc, Jackson, MS, USA).

Fruit production was monitored at monthly intervals, prior to each bat capturing, for all bat food plant species within the study sites, identifying and counting individual trees and shrubs that carried fruit. Following Wulms [38], food plants were identified along ten line-transects of approximately 80 m long, and a maximum detection distance between line-transects of 3 m. It was impossible to count the number of ripe fruits for certain tree species, especially for larger trees such as *Ficus* spp, so instead of counting fruits we recorded the number of plants with at least one mature fruit for all plant species. We quantified fruit availability as the number of shrub, trees and vines with fruits as potential food source for frugivorous bats per site. Fruit not encountered in fecal samples or carried by bats were not considered as part of the diet of frugivorous bats and eliminated from the analysis.

### Data analysis

We estimated the species diversity (Shannon *H*’; [39]) of frugivorous bats, fruit consumption and fruit availability using EstimateS [40]. The effect of forest type bat abundance was tested with separate General Linear Models (GLM), with forest type as fixed factor. We constructed residual plots to check for normality and log-transformed bat abundance to meet GLM requirements. Tukey post-hoc tests were performed on fixed factors if significant in the GLMs. 

We used a one-way ANOSIM [41] on Bray-Curtis similarity with Bonferroni corrected p-values to test whether frugivorous bat species composition and the composition of fruit species consumption differed among the forest types. 

To test for differences between the diversity of frugivorous bats, fruit consumption and fruit availability we used the two-tailed t-test proposed by Hutcheson [42]. To compare the species abundance of the five most abundant frugivorous bat species among forest types we tested the species abundance for normality using Shapiro-Wilk normality tests and log-transformed *C. sowelli* abundance and Box-Cox (Box and Cox 1964 [43]) transformed *C. perspicillata* abundance species abundance to meet GLM requirements. We tested for the effect of forest type on the abundance of both *Carollia* species with separate General Linear Models (GLM), including forest type as fixed factor, followed by Tukey *post-hoc* tests. Unlike *Carollia* abundance, both *Artibeus* species and *S. lilium* abundance presented a Poisson distribution and we therefore used a Generalized Linear Model (GZLM), followed by Bonferroni corrected Mann-Whitney U *post-hoc* tests, to test for the effect of forest type on the abundance of both species’ abundances [44].

We tested for the effect of forest type on fruit consumption per life form and successional stage with a Kruskal-Wallis test, due to non-normal data, followed by a Bonferroni corrected Mann-Whitney U post-hoc tests for non-parametric data. We were not able to normalize the fruit consumption per life form and successional stage, and therefore we tested for the effect of forest type on fruit consumption per life form and successional stage with a Kruskal-Wallis test, followed by a Bonferroni corrected Mann-Whitney U post-hoc tests for non-parametric data. 

Furthermore, we tested for differences in environmental variables, including structural variables (tree density, tree height, diversity of canopy trees and canopy openness) and fruit availability (fruit diversity and abundance) among forest types with an ANOVA, followed by Tukey post-hoc tests for normally distributed data, and a Kruskal-Wallis test, followed by a Bonferroni corrected Mann-Whitney U post-hoc tests for non-parametric data. All residuals were tested for normality using Shapiro-Wilk tests. All tests were performed in SPSS v17. 

We used the informative-theoretic approach [45], to model the bat diversity and abundance as dependent variables with environmental variables as independent variables, based on the second order Akaike’s Information Criterion corrected for small sample size (AIC_c_; i.e. n /K < ~40). The AIC_c_ values calculated from GLM’s has no meaning on its own, but comparing AIC_c_ estimations among different models is used as an approximation for the most meaningful model. Two measures associated with the AIC_c_ to compare the model fits were used; the delta AIC_c_ (Δ_*i*_) and Akaike weights (*w*_*i*_). The models returning an Δ_*i*_ < 2 are suggested to be the most meaningful models. Akaike weights (*w*_*i*_) give a measure of the relative strength of evidence, and indicate the weight of evidence in favour of being the best model. We used model averaging when not one of the models proved overwhelmingly supported by the data, by calculating the precision (SE) of the model-averaged estimate, termed as the unconditional SE, unconditional 95% confidence intervals (CI) and the relative importance of each independent variable [45]. Analyses on the informative-theoretic approach and model averaging were conducted in R v.3.0.0 [46].

Finally, we carried out a non-metric multidimensional scaling (NMDS) ordination, with Bray-Curtis as a measure of compositional dissimilarity [47], including abundance per bat species in relation to the differences in environmental variables per site, with 999 permutations, using the MetaMDS function in the R [44,46]. We used the stress value to measure the “goodness of fit” or the mismatch between distance measures and the distance in ordination space, with values smaller than 20 indicates a good ordination [48]. The final solution for the NMDS ordination after 999 permutations, was achieved within two runs of the data, with a stress value of 5.24 over a scale of 0 to 100.

## Results

### Frugivorous bat composition, diversity and abundance

**Figure 1:**
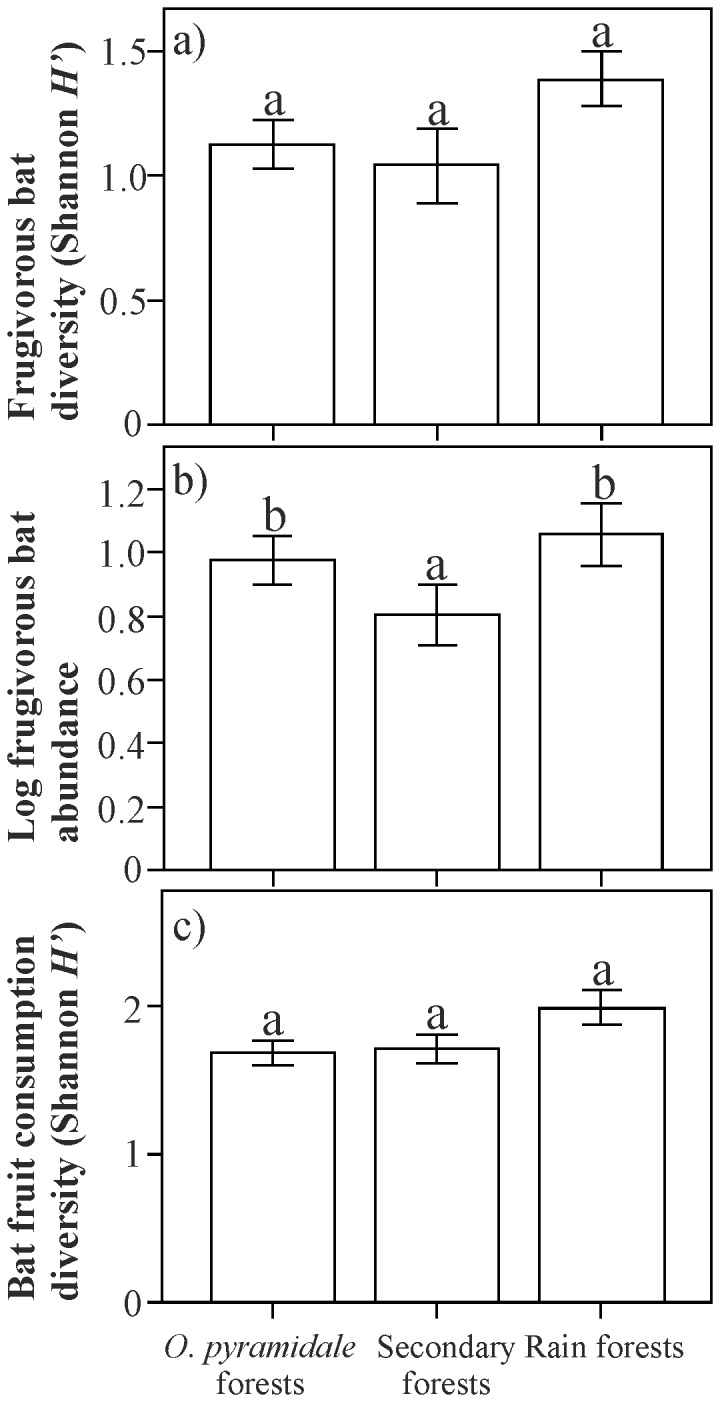
Comparisons of different parameters among forest types. Bars representing; (a) frugivorous bat diversity (Shannon *H*’), (b) log frugivorous bat abundance and (c) bat fruit consumption diversity (Shannon *H*’). Bars with equal letters are not significantly different; based on a two-tailed Hutcheson t-test for the frugivorous bat diversity and bat fruit consumption diversity and an ANOVA and Tuckey post-hoc test for the comparison of bat abundance.

A total of 1645 frugivorous bats, belonging to 18 species (Table 1), were captured and only two individuals were recaptured, with similar diversity (*H*’) between *O. pyramidale* forests and secondary forests (Hutcheson t-test t = -0.71, P > 0.05), *O. pyramidale* and rain forests (Hutcheson t-test t = -1.19, P > 0.05), and between secondary forests and rain forests (Hutcheson t-test t = -0.42, P > 0.05); Figure 1a). Bat abundance was affected by forest type, and was highest in *O. pyramidale* forests and in rain forests (F_2,141_ = 8.75, R^2^ = 0.110, P < 0.001; Figure 1b). The Anosim revealed a significant bat species compositional difference among forest types (global R = 0.1946, P < 0.001) and a pairwise comparison indicated significant differences between *O. pyramidale* forest and secondary forest (R = 0.1067, P < 0.01), *O. pyramidale* forest and rain forests (R = 0.2876, P < 0.01), and finally between secondary forest and rain forests (R = 0.1823, P < 0.01). 

**Table 1:** Number of captures per frugivorous bat species and subfamily in *Ochroma pyramidale* managed forests, secondary forests and rain forests.

**Subfamily**	**Species**	***O. pyramidale* secondary forest**		**Secondary forest**		**Rain forest**		**Total**	**% of total**
Carolliinae	*Carollia perspicillata*	185	**b**	112	**ab**	109	**a**	406	24.7
Carolliinae	*Carollia sowelli*	150	**b**	92	**a**	75	**a**	317	19.3
Stenodermatinae	*Artibeus jamaicensis*	19	**a**	28	**a**	171	**b**	218	13.3
Stenodermatinae	*Artibeus lituratus*	15	**a**	42	**a**	155	**b**	212	12.9
Stenodermatinae	*Sturnira lilium*	87	**b**	31	**a**	35	**a**	153	9.3
Glossophaginae	*Glossophaga commissarisi*	13		4		26		43	2.6
Glossophaginae	*Glossophaga soricina*	17		32		54		103	6.3
Phyllostominae	*Phyllostomus discolor*	0		0		12		12	0.7
Phyllostominae	*Phylloderma stenops*	0		0		1		1	0.1
Stenodermatinae	*Centurio senex*	0		1		3		4	0.2
Stenodermatinae	*Chiroderma villosum*	0		0		2		2	0.1
Stenodermatinae	*Artibeus phaeotis*	25		21		26		72	4.4
Stenodermatinae	*Artibeus toltecus*	4		4		4		12	0.7
Stenodermatinae	*Artibeus watsoni*	10		23		20		53	3.2
Stenodermatinae	*Platyrrhinus helleri*	4		5		8		17	1.0
Stenodermatinae	*Uroderma bilobatum*	8		0		7		15	0.5
Stenodermatinae	*Vampyressa thyone*	0		0		3		3	0.2
Stenodermatinae	*Vampyrodes caracciolii*	0		0		2		2	0.1
**Total**		**537**		**395**		**713**		**1645**	
**Total number of species**		**12**		**12**		**18**			

Differences in the number of captures of the five most abundant frugivorous bat species among forest types are shown in bold letters; based on a *post-hoc* Tukey test for *Carollia* species or *post-hoc* Mann-Whitney *U* test with Bonferroni correction for *Artibeus* bats and *Sturnira lilium*.

Comparison between most dominant bat species among the forest types revealed highest abundance of both *C. sowelli* (*F*_2,141_ = 9.40, *P* < 0.001) and *C. perspicillata* (*F*_2,141_ = 3.000, *P* = 0.053) in *O. pyramidale* forests, but a similar abundance of *C. perspcillata* in comparison with secondary forests (Table 1). Both *A. jamaicensis* (*χ*_2,141_ = 148.32, *P* < 0.001) and *A. lituratus* (*χ*_2,141_ = 111.204, *P* < 0.001) had higher abundances in rain forests, but their abundances in *O. pyramidale* forests and secondary forests was similar. *Sturnira lilium* abundance was highest in *O. pyramidale* and similar between secondary forests and rain forests (*χ*_2,141_ = 14.29, *P* = 0.001). 

### Fruit consumption by bats

Bats consumed fruits of a total of 27 plant morphological species (Table 2), of which 16 were identified to species level, three to genus level and two only to family level. We were unable to identify 6 seed species, which represented 6.5 % of the total fruit species consumed. The diversity of consumed fruit species (*H*’) was similar between the two secondary forest types (Hutcheson t-test t = -0.057, P > 0.05), *O. pyramidale* forest and rain forest (Hutcheson t-test t = 0.61, P > 0.05) as well as between secondary forest and rain forest (Hutcheson t-test t = -0. 54, P > 0.05; Figure 1c). The number of frugivorous bats that consumed fruits from early-successional trees (χ^2^_2,141_ = 3.32, P = 0.191) and late-successional shrubs fruits (χ^2^_2,141_ = 0.51, P = 0.773) was similar among forest type (Table 3). More bats that consumed fruits from early-successional shrubs were captured in *O. pyramidale* forests (χ^2^_2,141_ = 25.11, P < 0.001), and more bats consuming late-successional trees larger were captured in rain forests (χ^2^_2,141_ = 37.40, P < 0.001). The species composition of consumed fruits proved different among forest types (global R = 0.114, P < 0.001). Pairwise tests showed no difference in composition of consumed fruits species between *O. pyramidale* forests and secondary forests (R = 0.0224, P = 0.156), but did reveal a difference between *O. pyramidale* forests and rain forests (R = 0.1706, P < 0.001), as well as between secondary forests and rain forests (R = 0.1428, P < 0.001). 

**Table 2:** Number of plant species recorded from fruits consumed by frugivorous bats, successional categories and life form follows Greig [69], Guevara [70], Levy-Tacher and Aguirre-Rivera [71] and Pennington and Sarukhán [25].

**Species**	**Successional stage**	**Life form**	***O. pyramidale* secondary forests**		**Diverse secondary forests**		**Rain forests**	
			**N**	**%**	**N**	**%**	**N**	**%**
Brosimum alicastrum*	Mid/late	Tree	0	0.0	0	0.0	8	2.3
Calophyllum brasiliense var.j*	Late	Tree	0	0.0	0	0.0	2	0.6
*Cecropia obtusifolia*	Early	Tree	8	2.3	7	2.1	82	24.0
*Cecropia peltata*	Early	Tree	37	10.9	28	8.2	8	2.3
*Ficus americana*	Late	Tree	0	0.0	3	0.9	19	5.6
*Ficus maxima*	Late	Tree	2	0.6	4	1.2	16	4.7
*Ficus* spp 1	Late	Tree	3	0.9	0	0.0	4	1.2
*Ficus* spp 2	Late	Tree	0	0.0	0	0.0	13	3.8
*Piper aduncum*	Early	Shrub	83	24.3	38	11.1	21	6.2
*Piper aequale*	Mid/late	Shrub	1	0.3	1	0.3	0	0.0
*Piper aeruginosibaccum*	Mid/late	Shrub	16	4.7	35	10.3	6	1.8
*Piper auritum*	Early	Shrub	99	29.0	44	12.9	29	8.5
*Piper hispidum*	Early/mid	Shrub	35	10.3	28	8.2	25	7.3
*Piper umbellata*	Early	Shrub	8	2.3	7	2.1	2	0.6
*Piper* spp1		Shrub	2	0.6	0	0.0	3	0.9
*Prunus salicifolia*	Early	Tree	2	0.6	0	0.0	2	0.6
*Quararibea funebris*	Late	Tree	0	0.0	0	0.0	1	0.3
*Solanum erianthum*	Early	Shrub	34	10.0	6	1.8	8	2.3
*Solanum torvum*	Early	Shrub	2	0.6	0	0.0	4	1.2
Family Solanacea 1		Shrub	0	0.0	0	0.0	2	0.6
Family Solanacea 2		Shrub	0	0.0	0	0.0	1	0.3
*Spp* uni.1			0	0.0	0	0.0	4	1.2
*Spp* uni.2			0	0.0	0	0.0	1	0.3
*Spp* uni.3			2	0.6	0	0.0	0	0.0
*Spp* uni.4			3	0.9	0	0.0	0	0.0
*Spp* uni.5			4	1.2	6	1.8	1	0.3
*Spp* uni.6			0	0.0	0	0.0	1	0.3
**Total**			341		207		263	
**Total species**			16		12		23	

* Shows fruits only found carried by bats, i.e. not recorded from droppings.

**Table 3:** Mean abundance (± AAD; average of absolute deviation) as the number of frugivorous bats per seed species encountered in fecal samples or carried fruits categorized per life form (tree and shrub) and successional stage (early or late).

	***O. pyramidale* forests**				**Secondary forests**				**Rain forests**			
Early-successional trees	0.98	±	0.98	**a**	0.75	±	0.81	**a**	1.92	±	2.03	**a**
Early-successional shrubs	4.71	±	3.96	**b**	1.98	±	1.40	**a**	1.35	±	1.65	**a**
Late-successional trees	0.10	±	0.19	**a**	0.15	±	0.26	**a**	1.35	±	1.39	**b**
Late-successional shrubs	1.10	±	0.51	**a**	1.31	±	1.62	**a**	0.71	±	0.23	**a**

Differences in the number of frugivorous bats per life form and successional stage among the forest types are shown in bolds letters, based on Kruskal-Wallis and post hoc Mann-Whitney U test with Bonferroni correction.

### Forest structure and fruit availability

The forest structure differed among forest types (Table 4), with lower tree density (χ^2^_2,69_ = 36.30, P < 0.001) in rain forests and higher tree height (χ^2^_2,69_ = 22.83, P < 0.001) in *O. pyramidale* forests compared to secondary forests. The diversity of trees was similar between the forest types; *O. pyramidale* forests and secondary forests (Hutcheson t-test t = -1.24, P > 0.05), *O. pyramidale* forests and rain forests (Hutcheson t-test t = -1.38, P > 0.05), and between secondary forests and rain forests (Hutcheson t-test t = -0.51, P > 0.05). Canopy openness was highest in *O. pyramidale* forests (χ^2^_2,178_ = 135.33, P < 0.001).

**Table 4:** Vegetation structure, and monthly fruit diversity, abundance and availability of *Piper* and *Cecropia* species per ha, per forest type with *Ochroma pyramidale* forests, secondary forests and rain forests.

**Environmental variable**	***Ochroma pyramidale* forests**				**Secondary forests**				**Rain forests**			
Tree density (N/ha)	1450	±	3.39	**b**	1988	±	6.85	**c**	875	±	307	**a**
Tree height (m)	12.4	±	1.8	**b**	9.8	±	1.7	**a**	12.8	±	2.9	**ab**
Tree diversity (*H*')^	1.53	±	0.31	**a**	1.7	±	0.23	**a**	1.87	±	0.39	**a**
Canopy openness (%)	13.5	±	2.6	**c**	10.2	±	1.8	**b**	5.3	±	1.2	**a**
Fruit diversity (*H*')^	1.1	±	0.23	**a**	1.5	±	0.2	**a**	0.8	±	0.14	**a**
Fruit abundance (N/ha)*	76.9	±	47.8	**b**	102.0	±	57.8	**b**	18.3	±	10.2	**a**
*Piper auritum* (N/ha)	1.3	±	1.3	**a**	4.3	±	1.9	**b**				
*P. aduncum* (N/ha)	4.8	±	5.8	**a**	2.6	±	2.6	**a**				
*P. hispidum* (N/ha)	3.8	±	3.3	**a**	3.3	±	3	**a**				
*P. aeruginosibaccum* (N/ha)	0.54	±	0.9	**a**	2.7	±	4.2	**a**	1.4	±	2.3	**a**
*P. aequale* (N/ha)					0.77	±	1.4	**a**	1.4	±	2.4	**a**
*Cecropia obtusifolia* (N/ha)	1.5		1.5	**a**	2.9	±	2.8	**b**				

Letters in bold represent significant differences between treatments for each vegetation structure variable based on ANOVA and post hoc Tukey test (*), or Kruskal-Wallis and post hoc Mann-Whitney *U* test with Bonferroni correction and a Hutcheson two-tailed t-test (^). The difference in fruit availability of species only present in two forest types (*Piper auritum*, *P. aduncum*, *P. hispidum* and *Cecropia obtusifolia*) was tested with a Mann-Whitney *U* test.

Fruits in *O. pyramidale* forests and secondary forests were originated from only four shrub species (Table 4): *Piper auritum, P. aduncum, P. hispidum*, *P. aeruginosibaccum*, and one tree species *Cecropia obtusifolia*. *Piper aequale* was only found in secondary forests*. Ficus americana*, *F. maxima* and *Calophyllum brasiliense* were only reported in the rain forest, where also the late-successional shrub species *P. aeruginosibaccum* and *P. aequale* were found. 

Fruit availability of *P. auritum* (*Z*_1,94_ = -6.64, P < 0.001) and *C. obtusifolia* (*Z*_1,94_ = -0.57, P = 0.034) was higher in secondary forests than in *O. pyramidale* forests, but for the other *Piper* species, fruit availability was similar between secondary forest types. 

The diversity of available fruit species was similar between all three forests types (Hutcheson t-test t < 1.15, P > 0.05). Whereas the fruit abundance (χ^2^_2,36_ = 14.35, P = 0.001) was highest in both *O. pyramidale* forests and secondary forest, and lowest in rain forests.

### Predictors of bat diversity and abundance

The best model explaining the differences in bat species diversity included a negative relation with tree density and canopy openness, (Table 5). Bat abundance was best explained by a positive relation with fruit abundance and tree height, and a negative relation with tree density and canopy openness. 

**Table 5:** Best model selections using Akaike information criterion (AICc) approach and model averaging with bat diversity and (log) bat abundance as dependent variables and tree density, tree height, canopy and fruit abundance as independent variables.

Best model		Number of parameters	AIC_c_	Delta AIC_c_ (Δ_*i*_)	Akaike weight (*w*_*i*_)	Coeff	SE	95% CI		Relative importance
								2.5%	97.5%	
Bat diversity		2	160.46	0	0.14					
	Tree density					-0.022	0.009	-0.039	-0.005	0.92
	Canopy					-0.022	0.013	-0.049	0.005	0.57
(Log) Bat abundance		4	61.73	0	0.30					
	Canopy					-0.015	0.009	-0.035	0.003	0.60
	Fruit abundance					0.014	0.003	0.007	0.021	1.00
	Tree density					-0.016	0.007	-0.031	-0.003	0.87
	Tree height					0.039	0.015	0.009	0.071	0.94

Each model presented AIC_c_ values, Akaike weights (*wi*) and delta AIC_c_ differences (Δ_i_). Standardized model-averaged coefficients (Coeff), weighted unconditional standard errors (SE), 95% confidence intervals (95% CI) and relative importance are provided for each independent variable in the best-supported models.

### Frugivorous bat species abundance ordination

**Figure 2:**
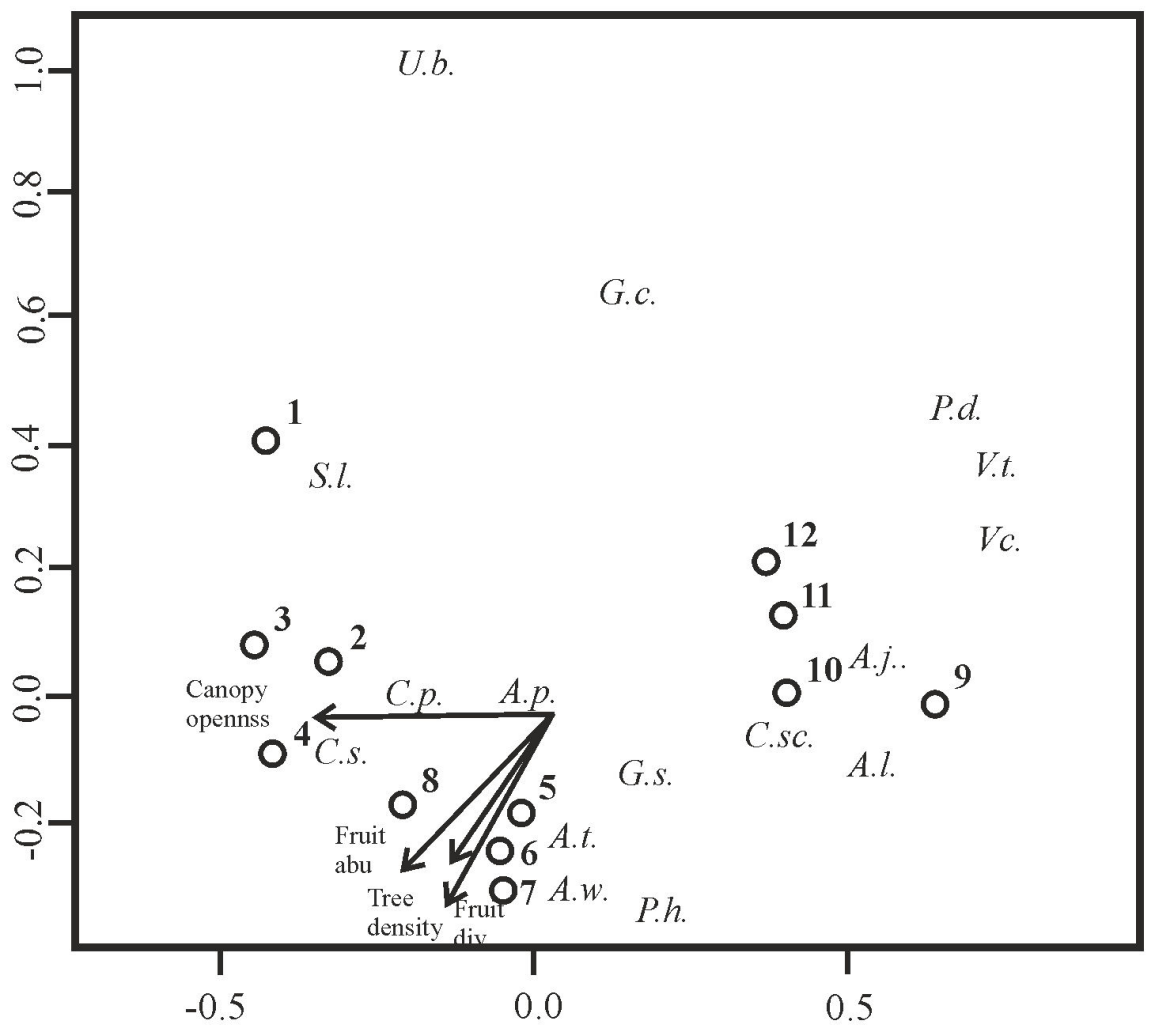
Non-metric multidimensional scaling (NMDS) ordination based on Bray-Curtis dissimilarity of frugivorous bat species abundance and the associated significant environmental variables. Sites: 1-4 *O. pyramidale* managed forests, 5-8 secondary forests and 9-12 rain forests. Directions of arrows indicate increasing variable value. Italic letters represent frugivorous bat species names, with 1st letter for genus and 2nd letter for species (exception: *Centurio senex* is *C. sx*; see Table 1).

The ordination clustered sites according to forest types, with a higher degree of similarity between *O. pyramidale* and secondary forest sites (Figure 2). Four of the six environmental variables (canopy openness, tree density, fruit abundance and diversity) proved significantly in the ordination (Table 6). The canopy openness seemed an important factor in separating *O. pyramidale* forests from secondary forest sites, with several characteristics bat species that tolerate higher canopy openness, such as *Carollia sowelli*, *C. perspicillata*, *Sturnira lilium* and *Uroderma billobatum* (Figure 2). Tree density, fruit abundance and fruit diversity were highest in secondary forests, and seemed negatively correlated to *Phylloderma stenops* and *Glossophaga commissarisi* species abundances, which were more common in rain forest.

**Table 6:** Significant structural vectors in the non-metric multidimensional scaling ordination using Bray-Curtis dissimilarity performed on frugivorous bat species abundance for each forest type site, showing correlation coefficient (r) and significance (*P*) based on 999 permutations.

**Environmental vector**	***r***	***P***
Canopy openness (%)	0.933	0.001
Tree density (N/ha)	0.654	0.009
Fruit abundance (N)	0.843	0.002
Fruit diversity (*H*')	0.684	0.007

## Discussion

### Bat composition, diversity, abundance and species-specific abundance

The management of secondary forests with the use of fast growing pioneer tree species *O. pyramidale* increases the abundance of frugivorous bats, equaling to that in rain forests. The bat species composition differed significantly among the forest types, with relatively more *Carollia sowelli* and *S. lilium* in *O. pyramidale* forests. These relatively small frugivorous bat species are probably attracted by the open canopy as seen from Figure 2, since they prefer early- to mid-successional secondary forests [49] for its abundance of fruit plants, such as the light demanding *Piper* spp. and *Solanum* spp. These plant species generally occur in a clumped distribution, but yield a constant year-round fruit production and therefore allow for food specialization of these bat species [50–53]. Even though the availability of *Piper auritum*, which is preferred by *Carollia sowelli* and generally forms an important part of its diet [54,55], was more abundant in secondary forests, this did not seem to cause a higher abundance of *Carollia sowelli* than in *O. pyramidale* forests. This suggests that other factors, such as structural vegetation characteristics like canopy openness might also be important for these bat species when searching for food. Larger frugivorous bats *Artibeus lituratus* and *A. jamaicensis* bats were abundantly present in rain forests, which provided them temporary but massive fruit availability of asynchronous, patchy distributed late-successional tree species [50,53]. Besides a preference for *Ficus* spp. [34], both large *Artibeus* bat species are also know for consuming *Cecropia* fruits [70]. The secondary forest patches with *Cecropia* fruit availability could function as an alternative source of food for both large frugivorous bat species in periods of low *Ficus* fruit abundance, but even though the fruit availability of *Cecropia* was higher in secondary forests in comparison with *O. pyramidale* forest, this did not result in a higher abundance of either *Artibeus* species. 

### Fruit availability and consumption

Managed *O. pyramidale* forests showed higher density of shrubs [11], but this did not result in higher fruit diversity or fruit abundance in comparison with other forest types. The composition of consumed fruits was significantly different between rain forests and both *O. pyramidale* forests and secondary forests, probably because various species of late-successional fruits that formed part of bat consumption (e.g. *Ficus* spp.) were only available in rain forests. This is confirmed by the fact that more bats consumed late-successional tree fruits in rain forests. Overall consumption consisted of mostly early-successional shrubs, which supports the fact that bats are known for their preference in early-successional shrub and tree fruits, and therefore have been indicated as facilitating successional development of early-successional areas [7,56]. As expected, secondary forests management with *O. pyramidale* increased the consumption of early-successional shrub fruits. Even though the diversity and abundance of fruits available in each of the secondary forest types was similar, the positive association between canopy openness and abundance of light demanding early-successional shrub species could be sufficient to attract more individuals of certain bat species such as *Carollia* and *Sturnira lilium*, with a preference for their fruits [7]. The consumption of late-successional fruits by bats in both secondary forest types was low, probably due to the absence of late-successional tree species and low abundance of large frugivorous bats, such as *A. lituratus* and *A. jamaicensis* that feed on these fruits. 

### Structural variables and fruit availability underlying differences in frugivorous bats

Both secondary and *O. pyramidale* forests, although with 10-15 years of vegetation succession, provided fruits from mostly early-successional and mid-to late-successional shrubs, with highest fruit diversity in secondary forests. However, only the abundance of fruits was positively correlated to the bat abundance, instead of fruit diversity, supporting the findings of an earlier study on increasing bat abundance with increasing fruit availability in agroecosystems [21]. Additionally, in areas of similar (temporal) fruit abundance, bat abundance and diversity is probably determined more by vegetation structural differences, such as tree density, height and canopy openness. In our study these variables were related with the bat assemblage structure, as also supported by other publications [18,21,57].

Tree density, previously reported to affect bat assemblages [58–60], was an important factor in the best models, negatively affecting both frugivorous bat diversity and abundance respectively. Higher densities of trees, as found in secondary forests [11], usually restricts the movement of bats, reducing their flight efficiency [61] and interferes with clutter echoes [62]. Tree height has been previously identified as an important characteristic related with bat roosts [63,64]. Tree height was highest in rain forest, offering suitable bat roosting sites [65]. 

Besides a lower tree density, *O. pyramidale* forests presented higher canopy openness than secondary forests, which was important in the second strongest model, negatively related to bat abundance. This is also found by previous studies [21,23,24,50,66], where bat movement of certain species, such as *Vampyressa thyone*, *Vampyrodes caracciolii* and *Phyllostomus discolor* was reduced due to lack of cover from predators [22] or because their preferred fruit species are generally not found under open canopies. As shown from the NMDS, the open canopy could positively affect the arrival of certain bat species such as *C. sowelli, C. perspicillata* and abundantly present in secondary forest types and tolerant towards areas of higher canopy openness, where light demanding fruit plants are more likely to occur. The relative open canopy in secondary forest dominated by *O. pyramidale* permits light to reach the understory, which is beneficial for the colonization of early-successional plant species and consequently provide food and shelter for generalist dispersers [67], as well as improving conditions favorable for the arrival and growth of mid-to late-successional plant species. However, without the reduction of canopy closure in *O. pyramidale* forests and the continuous dispersion of mostly early-successional shrub species by *S. lilium* and *Carollia* spp., the growth of late-successional plant species could be suppressed, delaying the successional development of these 10-15 year old forests [68]. 

We found that secondary forest management promoting trees of *O. pyramidale* affects the forests’ structural characteristics and fruit availability, and thereby alter the abundance of frugivorous bats, overall frugivorous species composition and affecting the movement of specific bat species such as *Carollia sowelli* and *S. lilium* towards more open *O. pyramidale* managed secondary forests. Hence, the application of forest management strategies can trigger cascading effects and consequently direct or change the speed of forest succession by affecting the arrival of important seed dispersers such as bats.
